# Supplementary material for: Deep level transient spectroscopic investigation of phosphorus-doped silicon by self-assembled molecular monolayers
Source: Nat Commun. 2018 Jan 9;9:118. doi: 10.1038/s41467-017-02564-3 (PMC5760684; doi:10.1038/s41467-017-02564-3)
Supplement: Supplementary file 1 — Supplementary Information [file 41467_2017_2564_MOESM1_ESM.pdf]

Supplementary information

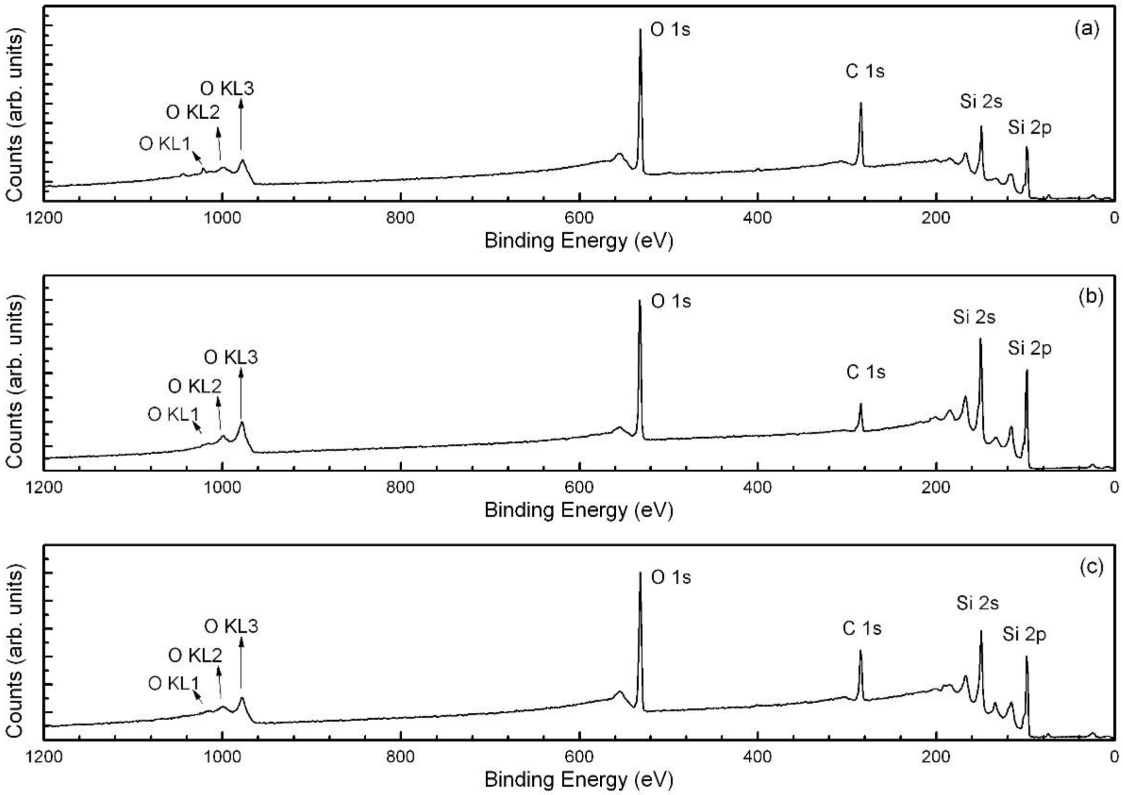

Supplementary Figure 1. XPS survey spectra of acetate-terminated surface (surface 2 in Figure 1) (a), hydroxyl-terminated surface (surface 3 in Figure 1) (b), and phosphorus-functionalized surface (surface 4 in Figure 1) (c).

Supplementary Note 1. Van der Pauw measurement

Sheet resistance was measured on square samples with Al electrodes on their corners.

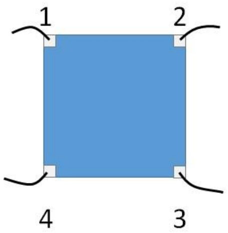

I-*V* curves measured along the input sides are shown in Supplementary Figure 2, demonstrating good ohmic contact of Al electrodes on the samples.

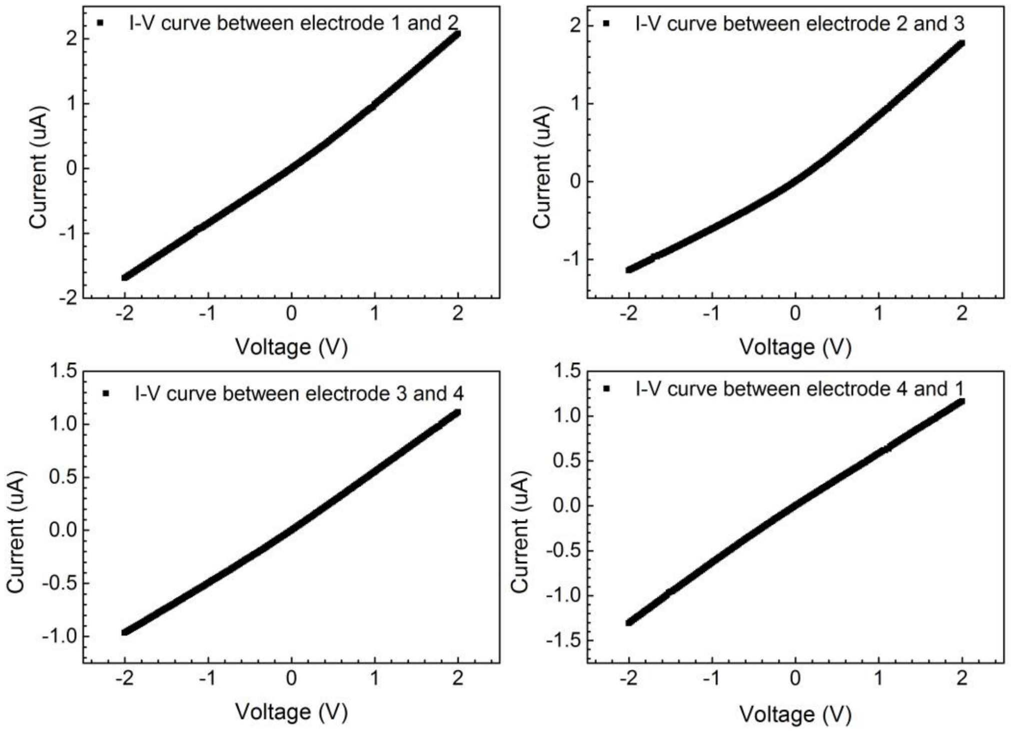

Supplementary Figure 2-a. *I-V* curves of the unmodified sample (as-received intrinsic-Si wafer after cleaning)

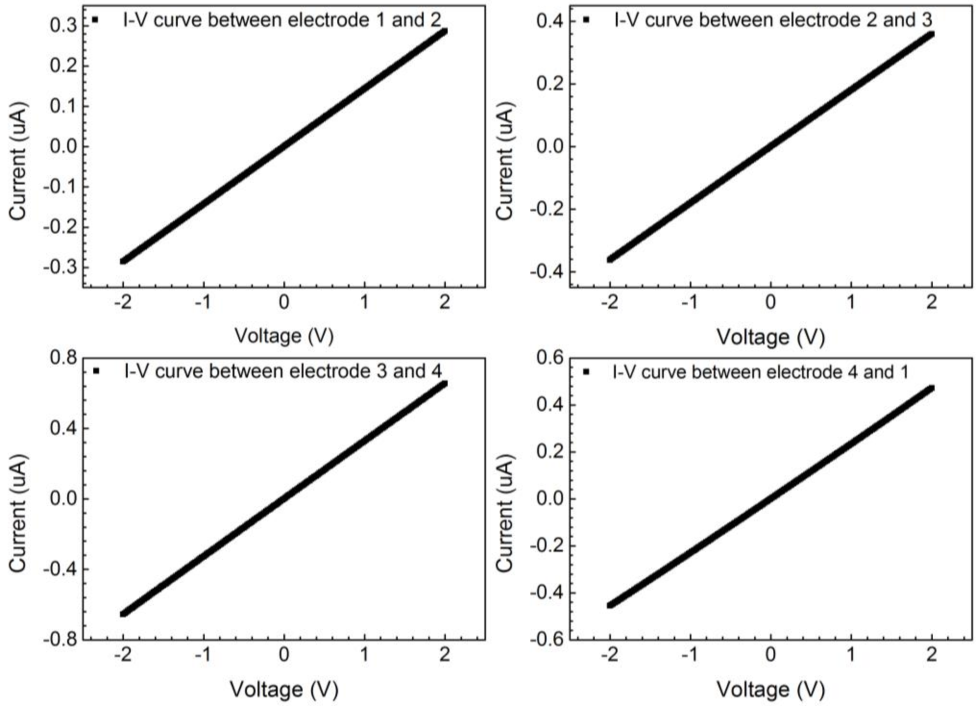

Supplementary Figure 2-b. *I-V* curves of the control sample (monolayer of carbon chains without phosphorus)

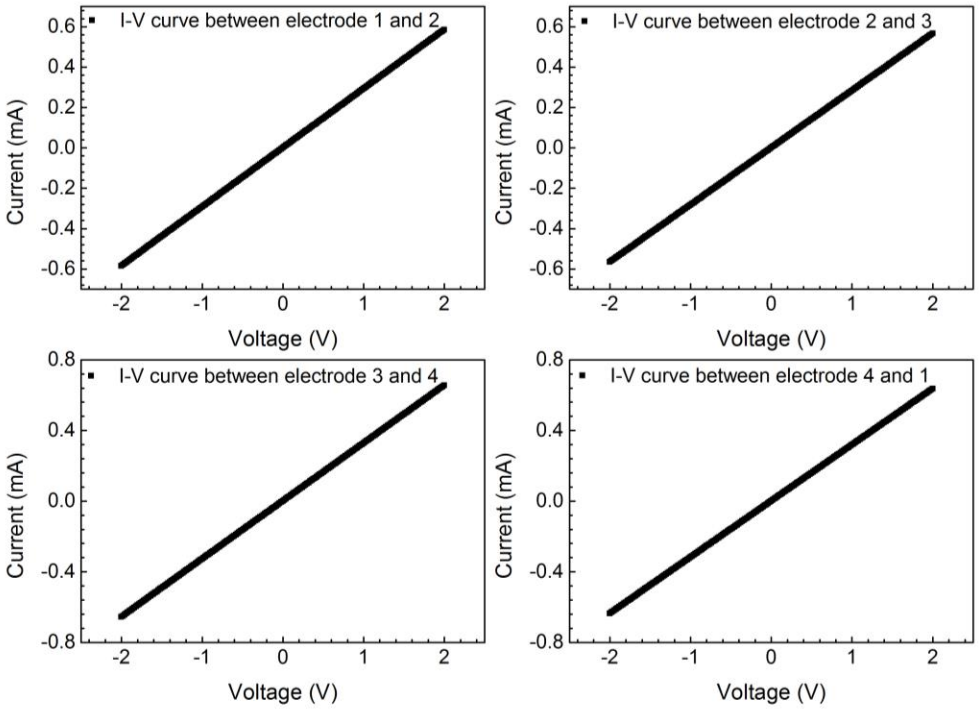

Supplementary Figure 2-c. *I-V* curves of the SAMM-doped sample

Supplementary Note 2. Notes for the first few data points in SIMS measurements

It is known that the first few points near the interface in SIMS data are usually inaccurate due to technical issue. In SIMS analysis, the element concentration is calculated according to the equation below:

$$C_{\mathrm{a}} = RSF \times \frac{I_{\mathrm{a}}}{I_{\mathrm{s}}}$$

where C_{a} is the concentration of element a, RSF is relative sensitivity factor, I_{a} is the intensity of ionized element a, I_{s} is the intensity of ionized substrate. For the first two or three steps of sputtering, the material is not pure silicon (for example, native oxide), but engineers usually use RSF of silicon to do calculation. In our case, since the dopants diffuse far into the substrates (tens of nanometers), only first few points have the error caused by this technical issue. The impact is thus negligible.

Supplementary Note 3. Uniform layer model versus multilayer model in Hall analysis

In the SAMM-doped silicon, phosphorus dopants distribute non-uniformly near the surface. For simplicity, electron concentration per unit area is calculated with a uniform layer model and the value is $8.92 \times 10^{12}\text{cm}^{-2}$. Ideally, the electron concentration derived from the multilayer model should be more accurate.[1] In the multilayer model, Hall coefficient is

$$R = \frac{te\int_0^t n(x)\mu^2(x)dx}{\left[e\int_0^t n(x)\mu(x)dx\right]^2} \tag{1}$$

where t is the doping depth, e the unit charge and $\mu(x)$ the charge carrier mobility. A widely used electron mobility model in silicon as a function of carrier concentration is shown in Supplementary Equation 2.[2,3]

$$\mu(x) = \mu_{\text{min}} + \frac{\mu_{\text{max}} - \mu_{\text{min}}}{1+\left(\frac{n(x)}{N_{\text{r}}}\right)^{\alpha}} \tag{2}$$

where $\mu_{\text{max}} = 1414\ \text{cm}^2\text{V}^{-1}\text{s}^{-1}$, $\mu_{\text{min}} = 68.5\ \text{cm}^2\text{V}^{-1}\text{s}^{-1}$, $N_{\text{r}} = 9.2 \times 10^{16}\ \text{cm}^{-3}$, $\alpha = 0.711$. The surface electron concentration is defined as：

$$N_{\text{e,m}} = \int_0^t n(x)dx \tag{3}$$

From Supplementary Equations 1 to 3, the electron concentration per unit area is found to be $N_{\text{e,m}} = 9.7 \times 10^{12}\ \text{cm}^{-2}$ at room temperature. It seems that a uniform layer model used before underestimates the electron concentration by ~ 8% in our case. However, empirical mobility Supplementary Equation 2 used in the multilayer model is derived from uniform doping in bulk silicon. In our research, phosphorus dopants distribute non-uniformly near the surface and are co-doped with carbon. Influence from surface and carbon makes it very difficult to ensure that the empirical mobility model (for bulk silicon) or the measured mobility values in literature accurately predicts (say less than 10% error) the actual mobility in our sample. The use of the mobility values that are of high uncertainty will only render even bigger uncertainty in the calculated electron concentration. It will be much more complex, in case of low temperature condition. Therefore, for simplicity we apply a uniform layer model, a small error of which will not change the conclusion in this research.

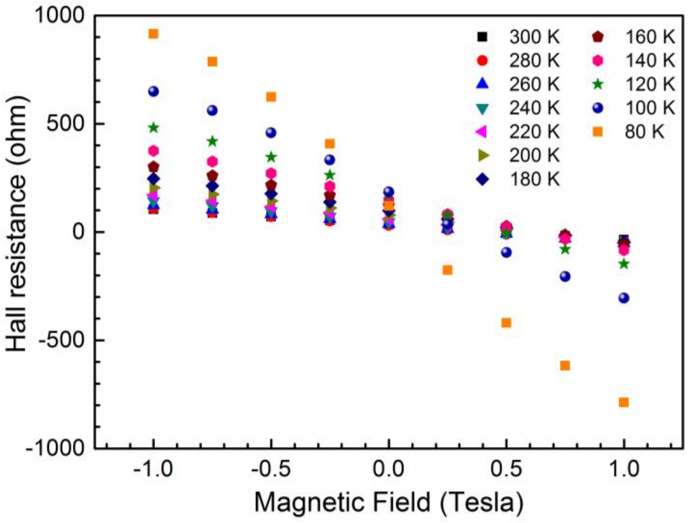

Supplementary Figure 3. Hall resistance versus magnetic field at different temperatures.

A series of Hall measurements were performed on the phosphorus-doped sample at temperature from 80 K to 300 K with an increase of 20 K, as shown in Supplementary Figure 3. The carrier concentration at different temperatures are calculated with Supplementary Equation 4, assuming a uniform doping layer. The results are listed in Supplementary Table 1.

$$N_{\mathrm{e}} = -\frac{B}{e \times (V_{\mathrm{H}}/I)} = -\frac{1}{e \times (slope)} \qquad (4)$$

where N_{e} is the electron concentration per unit area, e the unit charge, B the magnetic field and (V_{H}/I) the Hall resistance.

Supplementary Table 1. Carrier concentration at different temperatures derived from Hall measurements

Temperature	Slope	Carrier concentration
K	m^2C^{-1}	cm^{-2}
300	70.1	8.92×10^{12}
280	79.7	7.84×10^{12}
260	88.5	7.06×10^{12}
240	98.9	6.32×10^{12}
220	111	5.63×10^{12}
200	128	4.90×10^{12}
180	150	4.16×10^{12}
160	183	3.41×10^{12}
140	236	2.65×10^{12}
120	326	1.92×10^{12}
100	502	1.25×10^{12}
80	913	6.85×10^{11}

Supplementary Note 4. Ionization rate

To calculate the ionization rate of electrically active phosphorus (1.07×10^{13} cm^{-2}), an assumption is made that at each point of SIMS depth profile, 79.85% (=1.07/1.34) of the incorporated phosphorus is electrically active. Therefore, the distribution profile of this electrically active phosphorus (black curve in Supplementary Figure 4) is obtained by giving a factor of 0.7985 to the total phosphorus distribution by SIMS. When the phosphorus concentration is higher than 10^{17} cm^{-3}, only part of phosphorus is ionized, therefore a physical model of incomplete ionization by Andreas Schenk *et al.*[4] is employed, as Supplementary Equation 5 shows. Moreover, the internal electric field redistributes the ionized electrons, which in turn enhances the ionization of phosphorus dopants that are partially depleted. Therefore, a drift-diffusion system is built to simulate the influence of internal electric field, combined with the Poisson equation as shown in Supplementary Equations 6 to 8.

$$\frac{N_\mathrm{d}^+}{N_\mathrm{d}} = 1 - h\frac{n}{gn_1+n} \tag{5}$$

where g is the degeneracy factor and other parameters are:

$$h = \frac{1}{1+(\frac{N_\mathrm{d}}{N_\mathrm{b}})^r}$$

$$n_1 = N_\mathrm{c}\exp\left(-\frac{E_\mathrm{d}}{kT}\right)$$

$$\frac{\partial n}{\partial t} = \frac{1}{e}\nabla J_\mathrm{n} \tag{6}$$

$$J_\mathrm{n} = e(\mu_\mathrm{n} n \mathcal{E} + D_\mathrm{n}\nabla n) \tag{7}$$

$$\nabla^2\varphi = -\nabla\varepsilon = -\frac{e(p-n+N_\mathrm{d}^+-N_a^-)}{\varepsilon_\mathrm{r}\varepsilon_0} \approx -\frac{e(-n+N_\mathrm{d}^+)}{\varepsilon_\mathrm{r}\varepsilon_0} \tag{8}$$

in which n is the electron concentration, t the time, N_d the concentration of electrically active phosphorus, N_d^+ the concentration of ionized phosphorus, N_c the conduction band effective density of states, E_d the ionization energy, J_n the electron current density, μ_n the electron mobility, $\mathcal{E}$ the internal electric filed, D_n the electric diffusion coeffient, φ the electric potential, e is unit charge, k the Boltzmann constant, and T the temperature. For phosphorus doped silicon, N_b is 6×10^{18} cm^{-3},r is 2.3, and g is 0.5. [4]

At the equilibrium state ($\frac{\partial n}{\partial t} = 0$ and $J_\mathrm{n} = 0$), Supplementary Equation 9 is derived from Supplementary Equations 5 to 8. Hence, electron concentration n is influenced by both

incomplete ionization at high dopant concentration and internal electric field due to nonuniform doping.

$$\frac{q\cdot\mu_{\mathrm{n}}\cdot n}{\varepsilon_{\mathrm{r}}\varepsilon_0}\cdot(N_{\mathrm{d}}^{+}-\mathrm{n})+D_{\mathrm{n}}\frac{\partial^2 n}{\partial x^2}-D_{\mathrm{n}}\cdot\frac{1}{n}\cdot\left(\frac{\partial n}{\partial x}\right)^2=0 \qquad (9)$$

With arbitrary initial condition and boundary conditions below, the electron concentration is obtained as the red curve shows in Supplementary Figure 4.

Boundary conditions:

$$\left.\frac{\partial n}{\partial x}\right|_{x=\gamma}=0$$

$$\left.\varepsilon\right|_{x=\gamma}=0$$

where γ is equal to 0 or 300 nm.

Finally, ionization rate of electrically active phosphorus (1.07×10^{13} cm^{-2}) is 81.3%, as shown in Supplementary Equation 10.

$$ionizaton\ rate=\frac{\int n\, d_x}{\int N_{\mathrm{d}}\, d_x}=81.3\% \qquad (10)$$

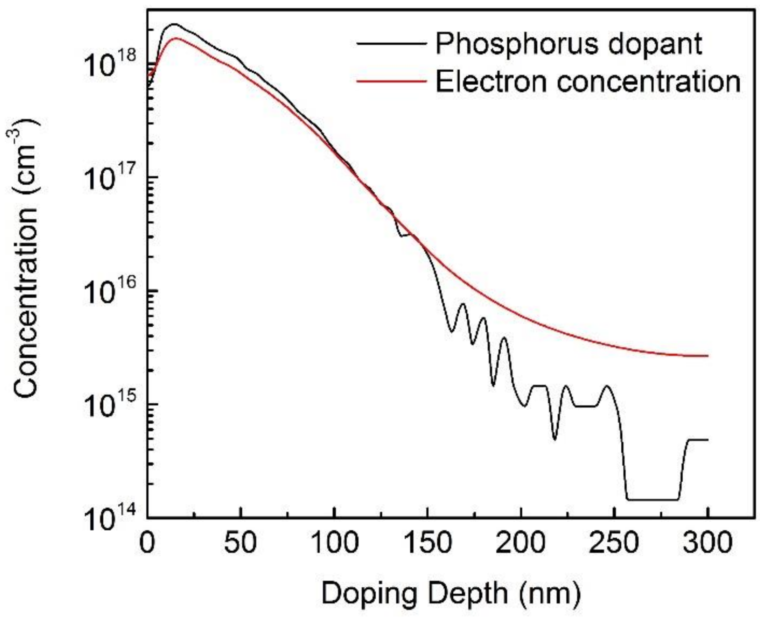

Supplementary Figure 4. Simulation results of electrically-active-phosphorus concentration versus doping depth (black curve) and electron concentration versus doping depth (red curve).

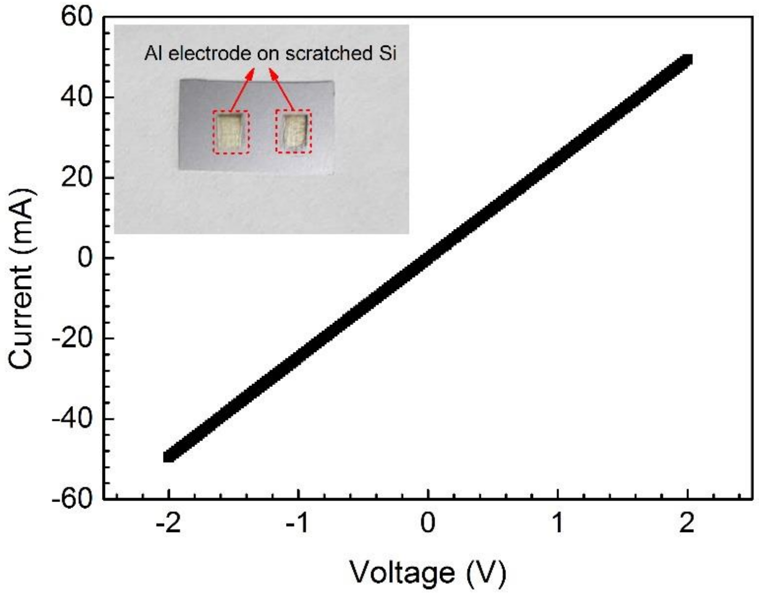

Supplementary Figure 5. *I-V* curve for Al electrodes on n-type Si wafer. Inset: optical image of the testing sample, on the backside of which two areas are firstly scratched and then deposited with Al electrodes after HF treatment.

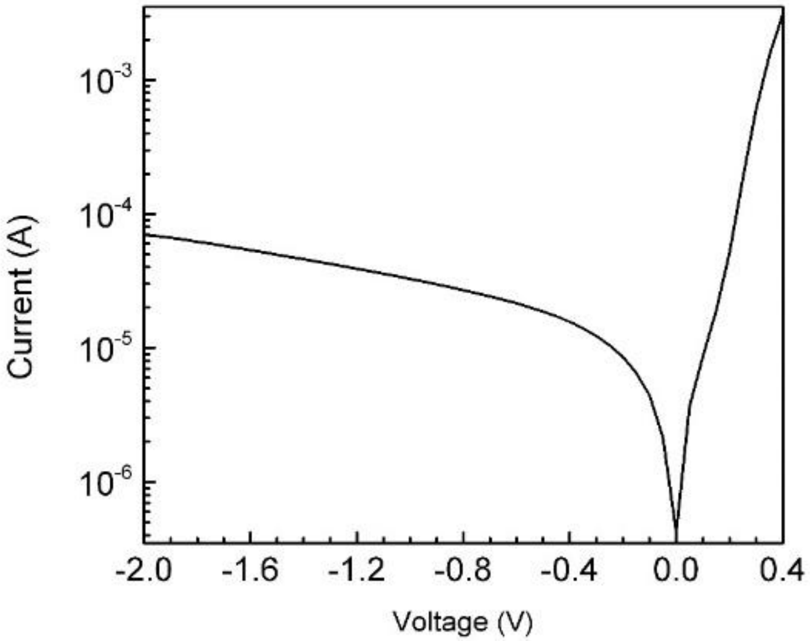

Supplementary Figure 6. *I-V* curve of the Schottky diode on the control sample.

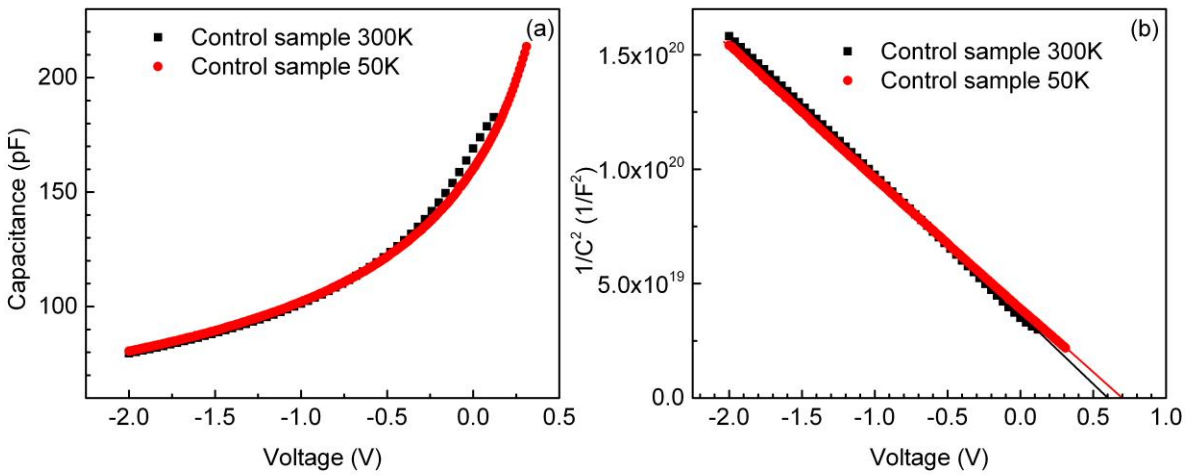

Supplementary Figure 7. *C-V* curves of the Schottky diode on the control sample. (a) *C-V* curves at 300 K and 50 K. (b) Dependence of $1/C^2$ versus voltage at 300 K and 50 K.

Supplementary Note 5. DLTS simulations

DLTS simulations are performed according to the following equation, which is transformed from Equations 3 and 4 in the manuscript. The experimental rate window in the case of the boxcar method is used in the present work in which the transient is sampled at two instant t_1 and t_2, with $t_2 > t_1$. A corresponding peak is detected when the trap's emission rate matches the rate window. That is the experimental emission rate $e_0=\ln(t_2/t_1)/(t_2 - t_1)$. When the rate window is 5 s^{-1}, t_1 is 0.122172 s and t_2 is 0.30543 s. When the rate window is 200 s^{-1}, t_1 is 0.003052 s and t_2 is 0.00763 s, *etc*.

$$\Delta C = \sum \Delta C_{0\mathrm{i}} \left(\exp\left(-\frac{2\sqrt{6} m \pi^{1.5} k^2 T^2 \, \sigma_{ni} \exp\left(-\frac{E_{\mathrm{ai}}}{kT}\right)}{h^3} t_1 \right) - \exp\left(-\frac{2\sqrt{6} m \pi^{1.5} k^2 T^2 \, \sigma_{ni} \exp\left(-\frac{E_{\mathrm{ai}}}{kT}\right)}{h^3} t_2 \right) \right) \tag{11}$$

Supplementary Note 6. Analysis of DLTS spectra

Deep energy levels can be calculated in Arrhenius plot with given emission rates and corresponding peak positions from DLTS spectra. Emission rate e_n is a function of temperature T, as shown in Supplementary Equation 12, where the average thermal velocity of electrons $< v_{\mathrm{n}} >$

and the effective density of states N_c are given by temperature-dependent functions as Supplementary Equations 13 and 14. Hence, the relation between emission rate e_n and temperature T can be written as Supplementary Equation 15. When plotted in logrithm scale, $\ln\left(\frac{e_\mathrm{n}}{T^2}\right)$ is linearly related to $\frac{1}{kT}$, and the slope gives the deep energy level, as shown in Supplementary Equation 16, which is applied in Arrhenius plot to derive deep energy level and capture cross section. The peak positions of the control sample and SAMM-doped sample at different rate windows (Figure 5a and b in the manuscript) are listed in Supplementary Table 2.

$$e_\mathrm{n} = (\sigma_\mathrm{n} < \nu_\mathrm{n} > N_\mathrm{c}/g)\exp\left(-\frac{E_\mathrm{a}}{kT}\right) \quad (12)$$

$$< \nu_\mathrm{n} > = \sqrt{\frac{3kT}{m}} \quad (13)$$

$$N_\mathrm{c} = 2\left(\frac{2\pi mkT}{h^2}\right)^{\frac{3}{2}} \quad (14)$$

$$e_\mathrm{n} = \frac{2\sqrt{6}m\pi^{1.5}k^2T^2\sigma_\mathrm{n}}{h^3}\exp(-E_\mathrm{a}/kT) \quad (15)$$

$$\ln\left(\frac{e_\mathrm{n}}{T^2}\right) = \ln\left(\frac{2\sqrt{6}m\pi^{1.5}k^2\sigma_\mathrm{n}}{h^3}\right) - \frac{E_\mathrm{a}}{kT} \quad (16)$$

where e_n is the emission rate, σ_n the capture cross section, $< \nu_\mathrm{n} >$ the average thermal velocity of electrons, N_c the effective density of states, g the degeneracy of the trap level, E_a the deep energy level, k the boltzmann constant and T the temperature.

Supplementary Table 2. Peak positions in DLTS spectra at different emission rates.

	Emission rate s^{-1}	Peak 0 (K)	Peak 1 (K)	Peak 2 (K)	Peak 3 (K)
Control sample	5	62.48	133.97	192.31	223.62
	10	64.49	136.88	198.06	231.37
	20	67.08	140.75	201.66	234.53
	50	69.52	146.93	209.40	245.30
	100	72.25	151.67	219.74	253.92
	200	76.13	157.84	224.48	259.23
SAMM-doped sample	5	133.00	158.13	223.33	242.00
	10	137.31	163.30	229.51	247.32
	20	141.47	167.47	235.68	254.78
	50	146.50	174.50	246.45	264.26
	100	151.96	179.10	255.07	273.74
	200	157.41	185.27	265.55	284.37

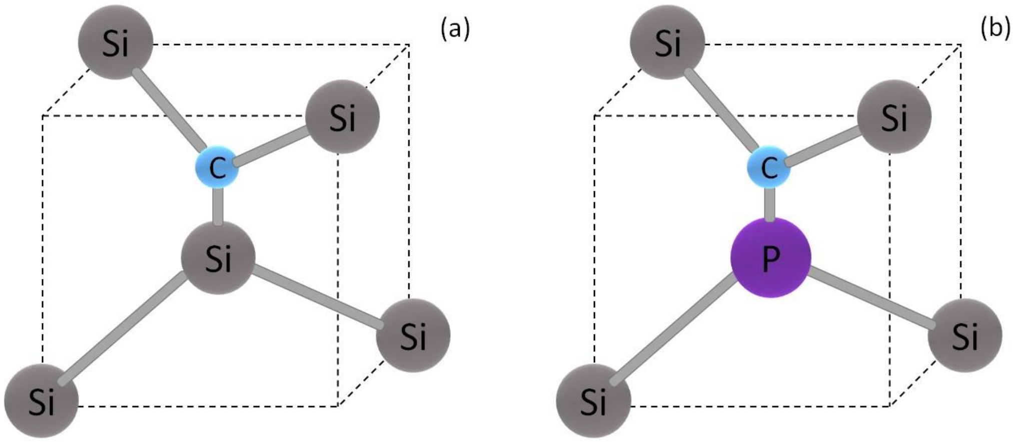

Supplementary Figure 8. Configurations of carbon interstitial (a) and C_i-P_s pair (b). [5]

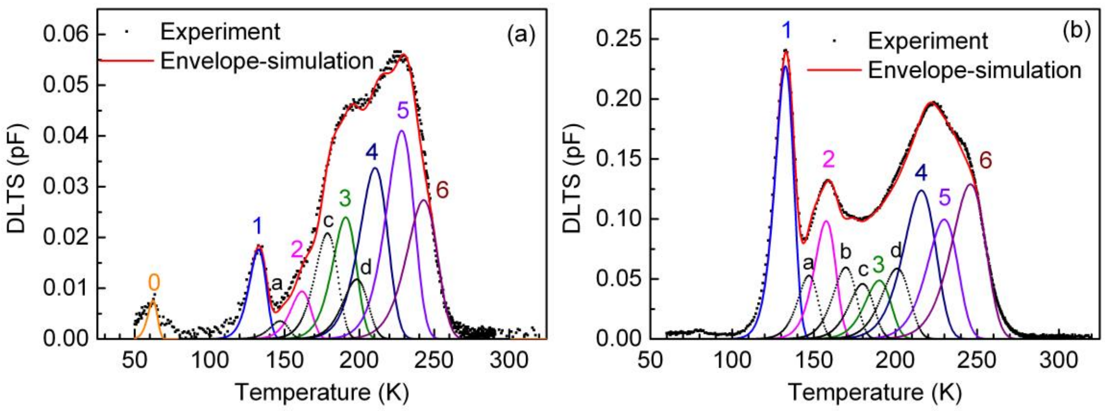

Supplementary Figure 9. Re-fitting of DLTS data of Figure 5e and Figure 5f as (a) and (b), respectively. The dotted peaks are the additional peaks.

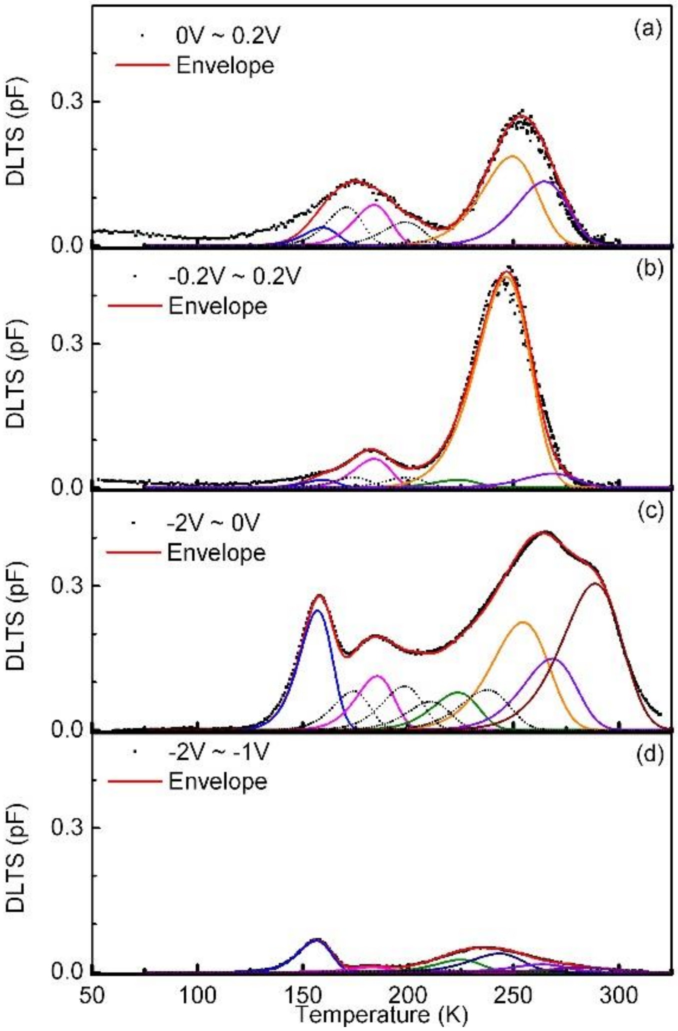

Supplementary Figure 10. Re-fitting of DLTS data of Figure 6c, e, g and i as (a), (b), (c) and (d), respectively.

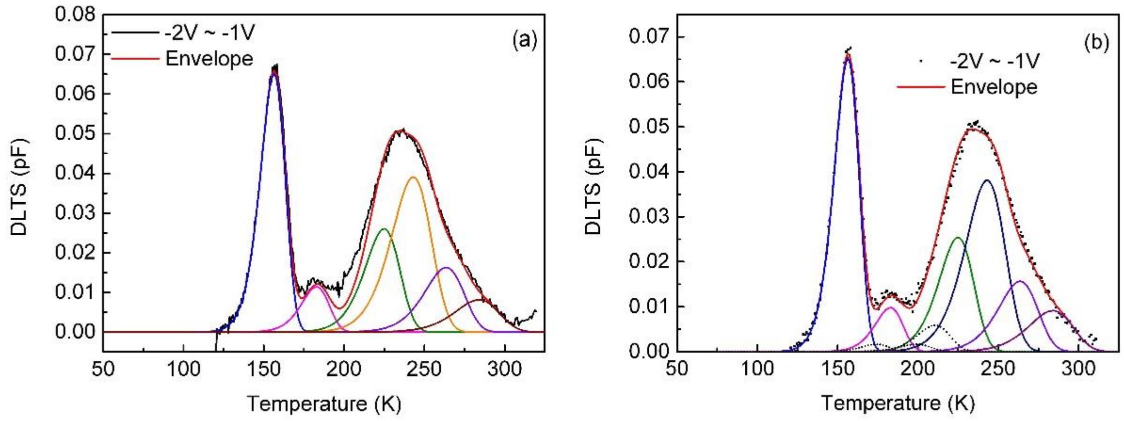

Supplementary Figure 11. Close-up plots of fittings for DLTS spectra of Figure 6i and Supplementary Figure 10d, as shown in (a) and (b), respectively.

Supplementary Table 3. List of energy levels derived from the extra fitting peaks in DLTS simulation.

	Bias pulse	Peak 0	Peak 1	Peak a	Peak 2	Peak b	Peak c	Peak 3	Peak d	Peak 4	Peak 5	Peak 6
Control sample	-2V ~ 0V	102	254	290	319	--	350	378	410	390	467	480
SAMM -doped sample	-2V ~ 0V	--	252	290	319	338	358	380	398	390	469	480
	-2V~ -1V	--	260	290	319	338	358	380	--	390	467	480
	-0.2V~ 0.2V	--	260	290	319	339	--	380	--	390	467	--
	0V ~ 0.2V	--	260	290	319	338	358	--	--	395	467	--

Extra peaks, peak a, b, c and d are indicated with underlines.

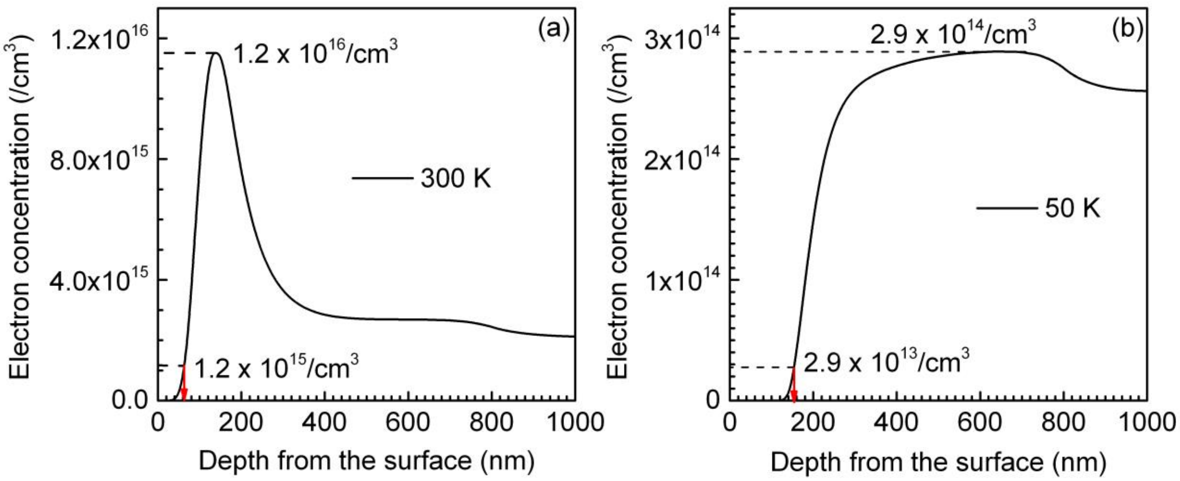

Supplementary Figure 12. Silvaco simulation on the electron distribution with a bias of 0.2V at 300K (a) and 50K (b). The red arrow is where the electron concentration is a tenth of the maximum. The left side of the red arrow is the depletion region.

To present the DLTS detection region clearly, Silvaco simulations on the band structure at 50 K were also conducted. The depletion width can be determined from the electron distribution profile (1/10 of the maximum) as shown in Supplementary Figure 12a and 12b.

As shown in Supplementary Figure 13b, when the bias was 0 V, the depletion region edge is located at 242 nm at 50 K (red dash line), and shifts to 100 nm as the temperature increases to 300 K. With an injection pulse of 0.2 V, the detection region width (grey area) can reach 64 nm at 300 K. Please see the depletion width in Supplementary Table 4 at other conditions.

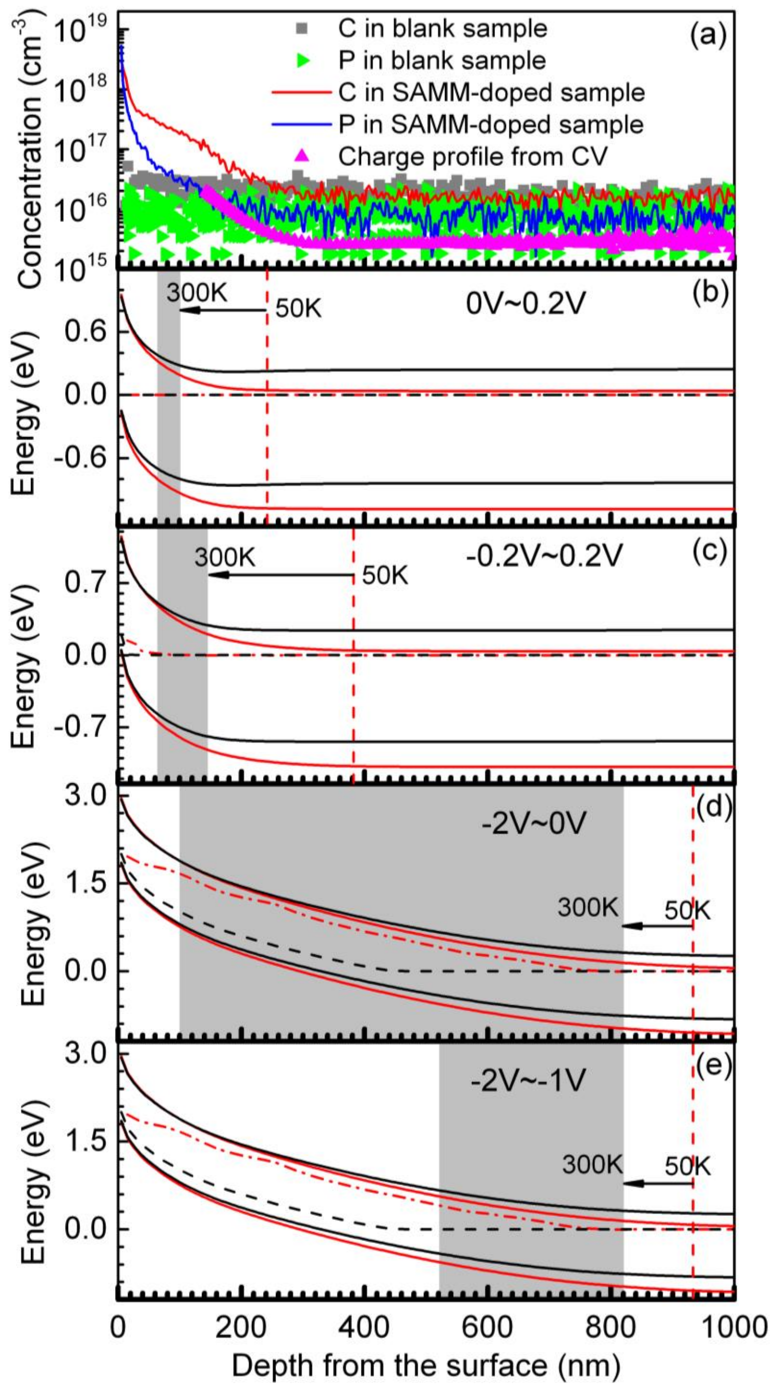

Supplementary Figure 13. P and C depth profiles by SIMS compared with ionized charge profile derived from *C-V* characteristics (a). Silvaco simulation on band structure at 300 K (black line) and 50 K (red line) with bias voltage of 0 V (b), -0.2 V (c), -2 V (d) and -2 V (e). Conduction band minimum and valence band maximum at 300 K are draw in black solid line, Fermi level at 300 K is in black dash line. Conduction band minimum and valence band maximum at 50 K are draw in red solid line, Fermi level at 50 K is in red dash-dot line.

Supplementary Table 4. Depletion width at different temperature and voltage bias.

Voltage	Depletion region width from surface		Voltage	Depletion region width from surface	
Bias	@ 300 K	@ 50 K	Pulse	@ 300 K	@ 50 K
0V	100 nm	242 nm	0.2 V	64 nm	154 nm
-0.2V	145 nm	382 nm	0.2 V	64 nm	154 nm
-2V	820 nm	933 nm	0 V	100 nm	242 nm
-2V	820 nm	933 nm	-1 V	522 nm	723 nm

In this table, the depletion width with voltage bias at a temperature of 50 K is shown with red dash line in Supplementary Figure 13. The depletion region with voltage bias at a temperature of 300 K is the right boundary of the grey area and pulse at 300 K is the left boundary of the grey area.

Supplementary References

1. Chien, C. L. & Westgate, C. R., The Hall Effect and Its Applications. 101-126 (Plenum Press, New York, 1980).

2. Caughey, D. M. & Thomas, R. E. Carrier mobilities in silicon empirically related to doping and field. *Proceedings of the IEEE* **55**, 2192-2193 (1967).

3. Ho, J. C. *et al.* Controlled nanoscale doping of semiconductors via molecular monolayers. *Nat. Mater.* **7**, 62-67 (2008).

4. Schenk, A., Altermatt, P. P. & Schmithusen, B. Physical model of incomplete ionization for silicon device simulation. in *International Conference on Simulation of Semiconductor Processes and Devices*, 51-54 (IEEE, 2006).

5. Zhan, X. & Watkins, G. Electron paramagnetic resonance of multistable interstitial-carbon–substitutional-group-V-atom pairs in silicon. *Phys. Rev. B* **47**, 6363 (1993).
